# COX-2 Is Downregulated in Human Stenotic Aortic Valves and Its Inhibition Promotes Dystrophic Calcification

**DOI:** 10.3390/ijms21238917

**Published:** 2020-11-24

**Authors:** Francesco Vieceli Dalla Sega, Francesca Fortini, Paolo Cimaglia, Luisa Marracino, Elisabetta Tonet, Antonio Antonucci, Marco Moscarelli, Gianluca Campo, Paola Rizzo, Roberto Ferrari

**Affiliations:** 1 Maria Cecilia Hospital, GVM Care & Research, 48033 Cotignola, Italy; vclfnc@unife.it (F.V.D.S.); frtfnc@unife.it (F.F.); paolocimaglia88@gmail.com (P.C.); m.moscarelli@imperial.ac.uk (M.M.); rferrari@gvmnet.it (R.F.); 2 Laboratory for Technologies of Advanced Therapies (LTTA), Department of Morphology, Surgery and Experimental Medicine, University of Ferrara, 44121 Ferrara, Italy; mrrlsu@unife.it; 3 Cardiovascular Institute, Azienda Ospedaliero-Universitaria di Ferrara, 44124 Cona, Italy; tonet.elisabetta@gmail.com (E.T.); tonio.antonucci@gmail.com (A.A.); cmpglc@unife.it (G.C.)

**Keywords:** CAVD (calcific aortic valve disease), AS (aortic stenosis), COX-2 (cyclooxygenase-2), AVICs (aortic valve interstitial cells), celecoxib, calcific nodules, calcification, aortic valve calcification

## Abstract

Calcific aortic valve disease (CAVD) is the result of maladaptive fibrocalcific processes leading to a progressive thickening and stiffening of aortic valve (AV) leaflets. CAVD is the most common cause of aortic stenosis (AS). At present, there is no effective pharmacotherapy in reducing CAVD progression; when CAVD becomes symptomatic it can only be treated with valve replacement. Inflammation has a key role in AV pathological remodeling; hence, anti-inflammatory therapy has been proposed as a strategy to prevent CAVD. Cyclooxygenase 2 (COX-2) is a key mediator of the inflammation and it is the target of widely used anti-inflammatory drugs. COX-2-inhibitor celecoxib was initially shown to reduce AV calcification in a murine model. However, in contrast to these findings, a recent retrospective clinical analysis found an association between AS and celecoxib use. In the present study, we investigated whether variations in COX-2 expression levels in human AVs may be linked to CAVD. We extracted total RNA from surgically explanted AVs from patients without CAVD or with CAVD. We found that COX-2 mRNA was higher in non-calcific AVs compared to calcific AVs (0.013 ± 0.002 vs. 0.006 ± 0.0004; *p* < 0.0001). Moreover, we isolated human aortic valve interstitial cells (AVICs) from AVs and found that COX-2 expression is decreased in AVICs from calcific valves compared to AVICs from non-calcific AVs. Furthermore, we observed that COX-2 inhibition with celecoxib induces AVICs trans-differentiation towards a myofibroblast phenotype, and increases the levels of TGF-β-induced apoptosis, both processes able to promote the formation of calcific nodules. We conclude that reduced COX-2 expression is a characteristic of human AVICs prone to calcification and that COX-2 inhibition may promote aortic valve calcification. Our findings support the notion that celecoxib may facilitate CAVD progression.

## 1. Introduction

Calcific aortic valve disease (CAVD) is the most common heart valve disease and the principal cause of aortic stenosis (AS) in Western countries. Angiotensin converting enzyme (ACE) inhibitors have shown some promise but their efficacy in counteracting the progression of AS in the clinical setting is still uncertain [1,2], while statins, although able to counteract calcification in vitro, were found to be ineffective in large randomized clinical trials [3,4]. Currently, there is no pharmacological therapy specifically indicated for CAVD, which progresses rapidly, eventually requiring the replacement of the aortic valve. CAVD is caused by fibro-calcific degeneration, which leads to the thickening and stiffening of valve leaflets. The cellular and molecular mechanisms underlying the onset and the progression of CAVD are only partially known.

During the progression of CAVD, aortic valve interstitial cells (AVICs) differentiate into myofibroblasts or osteoblasts, both contributing to the formation of calcific nodules [5]. Several studies in vitro and in vivo have shown that a number of inflammatory mediators promotes AVICs trans-differentiation, thus supporting the calcific degeneration [6]. Inflammation and oxidative stress cause endothelial dysfunction and decrease nitric oxide (NO) availability [7] playing an important role in the early stages of aortic valve disease [8]. Observations of increased levels of thioredoxin-interacting protein (TXNIP), which is suppressed by NO, in a rabbit model of mild aortic stenosis [9], and of increased levels of myeloperoxidase (MPO), a NO scavenger, in stenotic valves [10] are consistent with the hypothesis of an impairment of NO as a mechanism underlying AS [11,12,13]. On this basis, anti-inflammatory therapy has been considered as a possible strategy to interfere with CAVD progression [14].

Cyclooxygenase 2 (COX-2), which controls the synthesis of leukotrienes and prostaglandins, is an important mediator of inflammation and a target of commonly used non-steroid anti-inflammatory drugs. A high COX-2 expression was initially observed in AVICs in calcific nodules of Klotho mice, a model of early aging and aortic valve calcification [15]. In these mice, celecoxib, a selective COX-2 inhibitor, reduced aortic valve (AV) calcification [15]. However, in contrast with those findings, a recent retrospective clinical study revealed an association between AS and celecoxib use [16]. Noteworthy, in the same study, the pro-calcific effect of COX-2 inhibition was shown in vitro in porcine AVICs [16]. The role of COX-2 in AS has been mainly studied in non-human models and it remains uncertain whether COX-2 inhibition in human delays or promotes CAVD. In this study, we investigated COX-2 expression in human non-calcific and calcific AVs, and we explored the effect of celecoxib.

## 2. Results

### 2.1. COX-2 Expression in AVICs Is Decreased in Calcific Human Aortic Valves

**Figure 1:**
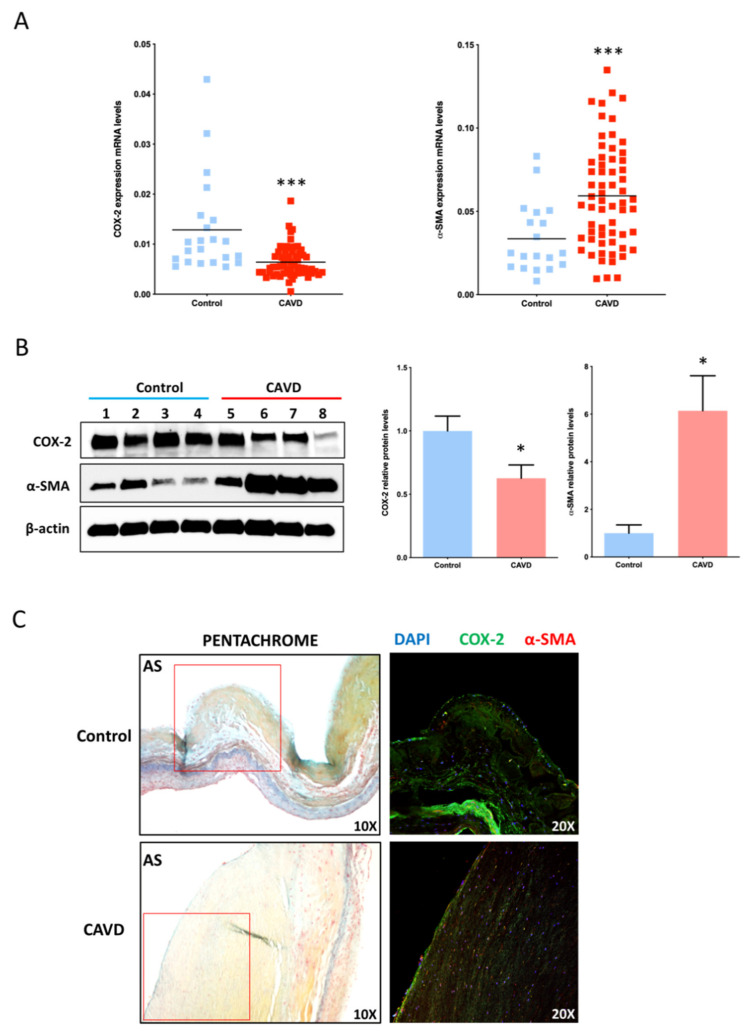
COX-2 expression is decreased in AVICs isolated from calcific aortic valves. (**A**) Cyclooxygenase 2 (COX-2) and α-smooth muscle actin (SMA) mRNA levels in non-diseased aortic valves (*n* = 22) and in calcific aortic valve disease (CAVD) (*n* = 61); *** *p* < 0.001 (**B**) Western blots of COX-2 and α-SMA in aortic valve interstitial cells (AVICs) isolated from non-calcific aortic valves (AVs) (*n* = 4) or calcified AVs (*n* = 4) and densitometric analysis of the levels of COX-2 and α-SMA of AVIC isolated from non-calcific AVs (*n* = 4) or calcified AVs (*n* = 4). Data were normalized for corresponding β-actin level. * *p* < 0.05 (**C**) Modified Movat’s pentachrome stain and immunohistochemistry staining of COX-2 (green) and α-SMA (red) in control or calcific AV. DAPI (blue) highlights cell nuclei. Images in the right panel show the corresponding areas indicated by red frames on the left panel. AS: Aortic Side.

To investigate the expression of COX-2 in CAVD, we quantified COX-2 mRNA levels in total RNA extracted from whole cusps of aortic valves from group 1 patients with CAVD and from those from group 2 without CAVD. Clinical characteristics of the two groups are reported in Table 1. We found that COX-2 mRNA levels were higher in non-calcific AVs compared with calcific AVs (0.013 ± 0.002 vs. 0.006 ± 0.0004 relative copy number; *p* < 0.0001) (Figure 1A). We then used linear regression modelling to investigate the effect of clinical variables on COX-2 expression. We found that AS (beta −1.037, 95% CI −1.479–−0.595, *p* < 0.001), age (beta −0.555, 95% CI −0.739–−0.371, *p* < 0.001) and LVMI (beta 0.288, 95% CI 0.017–0.559, *p* = 0.038) were associated to COX-2 levels at the univariable analysis. At multivariable analysis, AS (beta −0.695, 95%CI −1.238–−0.153, *p* = 0.013) and age (beta −0.436, 95% CI −0.675–−0.198, *p* = 0.001) were independently associated with COX-2 levels. This indicates that the reduction of COX-2 in CAVD occurs independently of age.

To further investigate the possible link between COX-2 levels and the progression of CAVD, we compared COX-2 levels with the levels of α-smooth muscle actin (SMA) in CAVD and control AVs. It is well known that a crucial role in AV calcification is played by activated fibroblasts, with properties of both fibroblasts and muscle cells, referred to as myofibroblasts, which originate from trans-differentiation of AVICs and are characterized by high levels of α-SMA [17]. As a result, in non-calcific valves, the majority of the AVIC population expresses low levels of α-SMA; conversely, in diseased AVs an increased number of α-SMA-expressing myofibroblasts is present [18]. Consistent with the presence of myofibroblasts in CAVD, we found that α-SMA expression was higher in calcific valves compared to non-calcific samples (0.034 ± 0.004 vs. 0.059 ± 0.005 relative copy number; *p* = 0.0005).

To investigate whether differences between CAVD and control AVs in COX-2 mRNA were related to the different expression of COX-2 in AVICs, we isolated AVICs from a subset of 4 controls and 4 CAVD samples and measured the levels of COX-2 protein. We found that AVICs from calcific valves have lower COX-2 levels, compared to non-calcific valves (Figure 1B). Additionally, as also found by others [19], AVICs from calcific AVs expressed higher levels of α-SMA than cells isolated from non-diseased valves (Figure 1B). Consistently, immunohistochemistry staining showed that non-calcified valves contain cells expressing COX-2 along with few α-SMA positive cells in an area including spongiosa and fibrosa identified by Movat’s pentachrome staining. The corresponding area of calcified samples is severely thickened and is populated by few COX-2 positive cells and several α-SMA positive cells (Figure 1C). Limited co-localization between COX-2 and α-SMA is present in controls while the few COX-2 positive cells in CAVD samples also express α-SMA, as shown by the presence of double positive (yellow/orange) cells (Figure 1C). Correlation analyses showed a negative association between α-SMA and COX-2 levels both in AVICs (R = −0.762; *p* = 0.02) and in AVs (R= −0.187; *p* = 0.05). Furthermore, COX-2 expression levels inversely correlated with the presence of CAVD (R= −0.858; *p* < 0.01). Taken collectively these data suggest that COX-2 reduction may play a role in CAVD by promoting the activation of AVICs.

In order to further dissect the role of COX-2 in the pathophysiology of CAVD, we performed correlation analyses between COX-2 expression levels and clinical, laboratory, and echocardiographic parameters in CAVD patients (Supplementary Table S1). No correlations were found between COX-2 levels and any of the parameters analyzed, including mean or aortic gradient or peak jet velocity, both linked to severity of CAVD (Supplementary Table S1). Consistently, we observed similar COX-2 levels in AVs with different degrees of calcification (Supplementary Figure S1). These data show that the downregulation of COX-2 is a characteristic of CAVD which does not progress with the worsening of the calcification and/or the disease.

### 2.2. Effect of Celecoxib in Nodule Formation of Human Aortic Interstitial Cells

**Figure 2:**
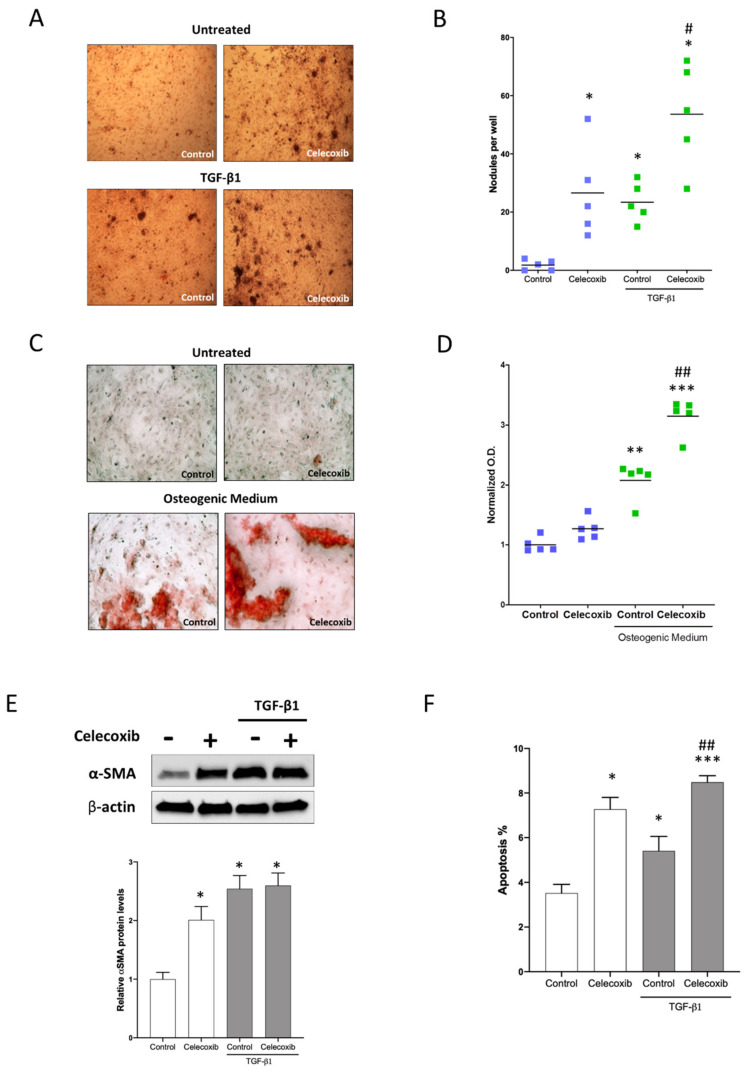
Celecoxib promotes myofibroblast induction, apoptosis and calcification in human AVICs. (**A**) In AVICs isolated from non-calcific AVs, TGF-β1 10 nM or celecoxib 5 µM induces the formation of calcium nodules (CN) identified by Alizarin Red staining. Images were taken using a 10× objective. (**B**) The experiments were performed with AVICs isolated from 5 non-calcific AVs. * *p* < 0.05 in comparison to control; # *p* < 0.05 in comparison to control with TGF-β1. (**C**) In AVICs isolated from non-calcific AVs and grown in osteogenic medium, celecoxib 5 µM induces the formation of calcium nodules identified by Alizarin Red staining Images were taken using a 20× objective. (**D**) Alizarin Red stain was quantified by acetic acid extraction followed by neutralization with ammonium hydroxide to enable colorimetric detection at 405 nm. The experiments were performed with AVICs isolated from 5 non-calcific AVs. ** *p* < 0.01, *** *p* <0.001 in comparison to control; ## *p* < 0.01 in comparison to control with TGF-β1. (**E**) Representative Western blot and densitometric analysis showing that TGF-β1 10 nM or celecoxib 5 µM induces the expression of α-SMA in human AVICs isolated from non-calcific AVs after 4 days. The experiments were performed with AVICs isolated from 3 non-calcific AVs. * *p* < 0.05 in comparison to control. (**F**) In AVICs isolated from non-calcific AVs, TGF-β1 10 nM or celecoxib 5 µM induces apoptosis. * *p* < 0.05, *** *p* < 0.001 in comparison to control; ## *p* < 0.001 in comparison to control with TGF-β1.

Since activation of the myofibroblast phenotype by TGF-β1 is considered a first step towards valvular disease [17], to explore the effect of COX-2 inhibition in CAVD, we induced the calcification of human AVICs isolated from non-calcific aortic valves by 4 days treatment with TGF-β1 [20] in the presence or absence of celecoxib and assessed the number of calcific nodules (CNs). As expected, treatment with TGF-β1 increased the number of CNs detected by Alizarin Red staining (Figure 2A,B). Importantly, in AVICs treated with celecoxib we observed an increased number of CNs, both in basal condition and in the presence of TGF-β1 (Figure 2A,B). Treatment with TGF-β1 strongly increased α-SMA protein levels in AVICs (Figure 2E) and celecoxib- only treatment induced α-SMA to an extent similar to that of TGF-β1 (Figure 2E). Differently from the effects of co-treatment on CNs, the addition of celecoxib did not further increase the level of α-SMA in comparison to treatment with TGF-β1 only. These data show that celecoxib induces activation of AVICs and promotes dystrophic calcification even in absence of TGF-β1. Furthermore, we investigated the effect of COX-2 inhibition in AVICs grown in osteogenic medium for 14 days. Under these conditions, AVICs display calcific accumulation as highlighted by Alizarin Red staining (Figure 2C,D). When celecoxib was added to the osteogenic medium, there was an increase in calcium accumulation, on the contrary when added to standard medium celecoxib alone did not increase calcification (Figure 2C,D). Finally, we measured apoptosis levels in AVICs following treatment with celecoxib and TGF-β1 for 4 days and observed that both TGF-β1 and celecoxib induce apoptosis in AVICs, the co-treatment with TGF-β1 and celecoxib has an additive effect on apoptosis (Figure 1F).

## 3. Discussion

We herein report that calcified human aortic valves have lower COX-2 expression in comparison to non-calcified valves. As expected, CAVD patients were older than patients without CAVD; however, multivariate analysis show that the reduction of COX-2 expression in calcific AVs occurs independently of age. In our study, together with reduced levels of COX-2 mRNA, we found increased levels of α-SMA in calcific valves. Importantly, this antagonistic relation between COX-2 and α -SMA was also evident in AVICs isolated from AVs of CAVD patients and non-calcific AVs. Overall, our findings are consistent with a mechanistic model of CAVD progression based on AVICs trans-differentiation into α-SMA expressing myofibroblasts, and additionally, show that these cells express less COX-2 in comparison to non-activated AVICs.

However, it must be considered that the inverse correlation between α-SMA and COX-2 levels in AVs samples was relatively weak. This could be due to several reasons: (i) dystrophic calcification involving increase of α-SMA and hence, trans-differentiation of AVICs into myofibroblast is expected to occur in about 80% of cases, the remaining 20% involves other mechanisms (i.e., trans-differentiation of a smaller population of AVICs into osteoblast-like cells driving ectopic bone formation) [21]; (ii) CD45-positive immune cells infiltrating AVs express COX-2 [15], hence the presence of active immune responses in AVs may be confounding factor; (iii) a portion of non-calcific AVs may have a certain degree of myofibroblast transition that could be clinically, and visually unnoticeable; (iv) individual differences, such as age and sex may affect the relative amount of different cell types, and consequently COX-2 and α-SMA levels.

In contrast with our data, COX-2 increase has been linked to CAVD based on the observation of a higher number of COX-2 expressing cells in human calcific valves, in comparison to healthy ones [15]. The different conclusions between our study and the study by Wirrig et al. could be due to the different approaches used to measure COX-2 levels (the number of COX-2 expressing cells in the proximity of the calcification versus the total levels of COX-2 in the valve). It should be also noted that the COX-2 expressing cells identified in the AV by Wirrig et al., were not inflammatory infiltrated cells and, similarly to our findings, did not express α-SMA. Since these cells were present in proximity of areas of calcification it could be that COX-2 upregulation represents an early event in the process of AVICs differentiation into myofibroblasts. In agreement with this hypothesis, it has been shown that interleukin (IL-1) stimulation has a biphasic effect on COX-2 mRNA levels with an initial increase at 2–4 h followed by a decrease below basal level after 24 h [22]. Based on this observation, it appears possible that inflammatory conditions in the aortic valve would first upregulate COX-2 expression in AVICs then leading to its decrease below a specific threshold triggering α-SMA upregulation and, thus, their trans-differentiation. Consistently with calcification occurring when COX-2 decreases below a specific value, we have not observed differences in the levels of COX-2 among AVs from patients with severe stenosis but different degrees of calcification. Our observation of reduced levels of COX-2 in calcific valve together with lack of correlation between levels of COX-2 and markers of gravity of CAVD, such as mean aortic gradient or peak jet velocity, may further indicate that COX-2 downregulation could be an early event that triggers CAVD.

However, we must consider that CAVD samples were all from patients with a severe stenosis; hence, we cannot rule out COX-2 reduction may occur later during the progression from an asymptomatic initial phase of the disease to severe stenosis that must be treated with valve replacement. Further studies correlating COX-2 levels with changes in peak gradient during the months leading to surgery should help to establish a possible role of COX-2 in the progression rate of aortic stenosis [23].

In agreement with the data in AVs, we found that in human AVICs isolated from non-calcific AVs, COX-2 inhibition with celecoxib promotes AVICs trans-differentiation towards a myofibroblast phenotype and the formation of calcific nodules, even in absence of TGF-β1, a multifunctional cytokine involved in collagen deposition and calcification of the AV [24]. Noteworthy, celecoxib also increased the levels of calcification measured by Alizarin Red when AVICs were grown in osteogenic condition. Importantly, our data on human AVICs mirrored data in porcine AVICs recently reported by Bowler et al., hence further strengthening observations in patients linking celecoxib to dystrophic calcification [16]. It is well known that cell death plays a crucial role in valve calcification [25,26]. In particular, apoptosis induced by TGF-β has been shown to be involved in dystrophic calcification pathways [27]. In this study, we found that celecoxib increases AVICs apoptosis, both in the absence or presence of TGF-β1. Overall, these findings indicate that celecoxib promotes myofibroblast trans-differentiation and the formation of early calcific foci containing apoptotic cells that may serve as a scaffold for further calcification.

We did not further explore molecular mechanisms by which COX-2 downregulation or inhibition by celecoxib promote myofibroblasts phenotype. COX-2 main product is prostaglandin E2 (PGE2) which has been shown to prevent and reverse myofibroblast differentiation [28]; recently, a study found that celecoxib also affects the osteogenic transition of porcine AVICs and the effect of celecoxib depends on the presence of glucocorticoids [29]. Interestingly, inhibition of prostaglandin synthesis by glucocorticoids has been reported [30]. It is also worth mentioning that the animal model in which celecoxib prevents calcification has been reported to have lower levels of glucocorticoids [29]. Given the crucial role, this should be considered when analyzing the activity on celecoxib in different models.

Interactions between drugs and CAVD have been investigated by others, both in the attempt to identify drugs that may promote CAVD or to identify possible strategies to slow down calcification. Association between aspirin and aortic valve calcification has been reported [31] and there is evidence that warfarin accelerates calcification by affecting matrix GLA protein activation [1,32]. On the contrary, ACE inhibitors or angiotensin receptor blockers (ARBs) may slow the progression of AS [33].

Most selective COX-2 inhibitors have been retired because of increased cardiovascular events; celecoxib, on the contrary, maintained its approval after a further safety investigation which did not revealed increased cardiovascular events compared to naproxen or ibuprofen [34] and it is currently indicated for a number of diseases including osteoarthritis, rheumatoid arthritis and juvenile arthritis. However, in this study, celecoxib safety was investigated only for relatively short terms events [34].

Our data indicate that reduced COX-2 expression is a characteristic of calcified aortic valves and show that COX-2 inhibitor celecoxib promotes a pro-calcific phenotype in AVICs isolated from human AV as previously shown in porcine AVICs [16].

This study has several limitations. First, in aortic valves samples we measured COX-2 as mRNA levels but we did not directly evaluate COX-2 activity. While COX-1 is constitutively expressed, COX-2 is an inducible enzyme. COX-2 mRNA and COX-2 protein exhibit shorter half-lives than those of COX-1. Hence, the COX-2 protein is only present for few hours after its synthesis, while it is tightly regulated transcriptionally [35]. For this reason, it seems reasonable to assume that COX-2 expression largely determines its activity. Importantly, although we did not measure COX-2 activity, we did observe that specific blocking COX-2 activity with celecoxib promotes calcification. Second, even if data in AVICs from diseased valve show that COX-2 inhibition promotes calcification, and we found a strong decrease of COX-2 expression in CAVD samples, it remains to be established whether the reduction of COX-2 expression is a cause or a consequence of CAVD. Future studies in which we will compare COX-2 expression in calcific valves and progression of the disease as determined by 6-month change in mean gradient in preoperative echocardiograms should provide further details on the role of COX-2 in this context [23]. Third, the control aortic valves come mainly from patients with aortic insufficiency. Although specimens with significant thickening or calcification nodules greater than 2 mm were not included in the study, some minor calcifications may be present. Fourth, our study included some bicuspid valves which are known to be susceptible to inflammatory activation, endothelial dysfunction and, consequently, calcification [36]. However, due to the limited number of samples, we were not able to determine whether COX-2 inhibition plays a role in this subset.

Additional studies are needed to confirm the link between celecoxib use and the development or progression of CAVD, as this could influence prescribing patterns for medications that relieve inflammatory pain. Of note, aortic stenosis is associated with gastrointestinal bleeding [37]; in principle, COX-2 inhibitors may be useful, as they have been developed for limiting gastrointestinal side effects, but they should be avoided if they exacerbate AV calcification.

## 4. Materials and Methods

### 4.1. Clinical Data

We studied 83 consecutive patients from March 2018 to July 2019 undergoing cardiac surgery at Maria Cecilia Hospital, Cotignola (RA), Italy; 61 patients had valve replacement for calcific severe aortic stenosis (group 1); 22 patients without AV calcification (group 2) had valve replacement for one of these conditions: (1) severe aortic insufficiency; (2) aneurysm of the aortic root. AV specimens from group 2 were considered non-calcific when no significant calcification was detected by previous echocardiogram and visual observation. Specifically, AVs with nodules of less than 2 mm in the absence of visible thickening were considered non-calcific. The study was approved by Ethics Committee of “Romagna” (approved on 26 January 2018, approval code: 590/2017) and was conducted according to the Declaration of Helsinki. All patients gave written informed consent.

### 4.2. Gene Expression

Aortic valves leaflets removed during surgery were immediately immersed in RNAlater (Thermo Fisher Scientific, Waltham, MA, USA). RNA was extracted from AV specimens. RT-PCR was performed as described in Supplementary Methods.

### 4.3. AVIC Isolation and Cell Culture

Human AVICs were isolated from aortic valves leaflets by two enzymatic digestions following established procedures with minor modifications [38,39]. Characteristics of donors are reported in Supplementary Table S2. Detailed procedures are described in Supplementary Methods.

### 4.4. Histology and Immunohistochemistry

Aortic valves removed during surgery were immediately fixed in 10% neutral buffered formalin for 24 h and then embedded in paraffin. For histology and immunostaining, 5-µm sections were cut using a microtome (SLEE medial, Mainz, Germany). Movat pentachrome stain (modified Russell-Movat) (Abcam, Cambridge; UK) was performed according to manufacturer’s instructions. For immunofluorescence analysis, after antigen retrieval slices were incubated overnight with an anti-COX-2 and anti-α-SMA antibodies and then washed and incubated with Alexa fluor-conjugated secondary antibodies. Immunofluorescence images were taken with a confocal microscope (Nikon A1 system) using 20X objective. Pentachrome staining images were taken with a microscope (Nikon Eclipse Ni) using a 10X objective. Procedures are described in detail in Supplementary Methods.

### 4.5. Western Blots

AVICs were collected by scraping and lysed on ice in RIPA buffer (Thermo Fisher Scientific). Western blots were performed as previously described [40]. Antibodies used are listed in Supplementary Methods.

### 4.6. Nodule Formation Assay

AVICs were plated onto plates or Petri dishes at a density of 75,000 cells/cm^2^ in M199 medium supplemented with 1% FBS, with or without 5 ng/mL recombinant human transforming growth factor (TGF)-β1 (Sigma Aldrich, St. Louis, MO, USA) [20] and with or without 5 µM celecoxib (Sigma Aldrich). After 4 days, cells were fixed for 10 min in 4% paraformaldehyde (PFA, Sigma Aldrich). Finally, cells were rinsed with deionized water, incubated with 14 mM Alizarin Red Stain (Sigma Aldrich) for 10 min. Cells were washed 2 times with PBS to remove excess of Alizarin Red. Round/oval and red-stained nodules with a diameter of 50–100 µm (estimated from 5-cell diameter) [20]. Red-stained nodules were manually counted under an inverted microscope (Nikon). Alizarin Red was also quantified from the stained cells by acetic acid extraction followed by neutralization with ammonium hydroxide to enable colorimetric detection at 405 nm using microplate photometer (Multiskan, ThermoFisher Scientific).

### 4.7. Apoptosis

Apoptosis was determined with the Annexin V binding assay. AVICs were stained with Annexin V-FITC (Thermo Fisher Scientific) and propidium iodide (Sigma-Aldrich), as described in details in Supplementary Methods.

### 4.8. Statistical Analyses

Normality was assessed before further analysis. Student t test for independent measures was employed to test statistical significance in the mean differences of gene expression levels in two groups. Linear regression modelling was used to analyze the effect of clinical variables on COX-2 values. All baseline variables were tested in a univariable model, and those found to be significant (*p*-values ≤ 0.05) were included in adjusted multivariable linear regression analysis. Results are reported as standardized beta with associated 95% confidence intervals (CI). The multicollinearity was examined using the variance inflation factor (VIF) and variables with VIF > 5 were excluded by the same multivariable model. Variable selection was performed by a backward stepwise algorithm based on Akaike’s information criterion minimization. Correlations between continuous or dichotomous variables were tested by Spearman’s correlation coefficient. ANOVA applying appropriate post-test for multiple comparisons were used to compare groups of in vitro experiments. *p*-values ≤ 0.05 were considered statistically significant. Statistical analysis was performed with GraphPad Prism version 8.0 (GraphPad software Inc., San Diego, CA, USA) or with SPSS Statistics version 26.0 (IBM, NY, USA).

## Figures and Tables

**Table 1:** Clinical characteristics.

	CAVD (*n* = 61)	Control (*n* = 22)	*p*
Age (years)	78 (73–81)	71 (64–78)	0.002
Male sex	33 (54)	15 (68)	0.251
BMI (kg/m^2^)	27 (24–29)	28 (25–30)	0.274
Bicuspid	4 (6.5)	2 (11.0)	0.653
**Medical history**			
Hypertension	54 (89)	22 (100)	0.181
Dyslipidemia	41 (67)	14 (64)	0.761
Diabetes	13 (21)	1 (5)	0.099
Smoke – Never – Prior – Current	32 (53) 24 (39) 5 (8)	7 (32) 9 (41) 6 (27)	0.052
Severe CAD	9 (14)	2 (9)	0.719
Prior PCI	6 (10)	2 (9)	1.000
Prior stroke	2 (3)	1 (5)	1.000
PAD	11 (18)	4 (18)	1.000
AF	5 (8)	8 (36)	0.004
COPD	9 (15)	3 (14)	1.000
CDK	32 (63)	9 (43)	0.694
**Drug therapy**			
Warfarin	6 (10)	10 (45)	<0.01
ASA	30 (49)	12 (54)	0.804
**Laboratory data**			
Hemoglobin (g/dL)	13 (12–14)	14 (12–15)	0.537
Platelets (×10^3^/mm^3^)	207 (177–246)	176 (155–191)	0.069
Glucose (mg/dL)	106 (93–120)	97 (90–113)	0.407
eGFR (mL/min)	59 (49–72)	61 (44–74)	0.771
LDL (mg/dL)	94 (70–119)	99 (77–119)	0.513
Albumin (g/dL)	4.3 (4.1–4.5)	4.3 (4.1–4.5)	0.707
**Echocardiography data**			
LVEDVi (mL/m^2^)	51 (42–62)	76 (50–98)	0.004
LV ESVi (mL/m^2^)	19 (16–26)	28 (15–43)	0.048
LV EF (%)	61 (51–68)	65 (50–69)	0.613
LVMI (g/m^2^)	118 (102–139)	135 (91–187)	0.410
AV MPG (mmHg)	47 (41–57)	8 (7–25)	<0.001
AV peak velocity (m/s)	4.4 (4.1–4.8)	2.0 (1.7–3.2)	<0.001
Significant AR	6 (10)	17 (77)	<0.001

Continuous variables are presented as median (interquartile range), categorical variables are presented as count (percentage). BMI, body mass index; PCI, percutaneous coronary intervention; CAD coronary artery disease; PAD peripheral artery disease; AF, atrial fibrillation; COPD, chronic obstructive pulmonary disease; CDK, chronic kidney disease; ASA, acetylsalicylic acid; eGFR, estimated glomerular filtration rate; LDL, low density lipoprotein; LV, left ventricle; EDVi/ESVi, end diastolic/systolic volume index; EF, ejection fraction; LVMI, left ventricle mass index; AV, aortic valve; MPG, mean pressure gradient; AR, aortic regurgitation. Comparisons between groups were performed with independent sample *t*-test, Mann–Whitney U-test, Pearson’s Chi-squared test, or Fisher’s exact test, as appropriate.

## Supplementary Materials

Supplementary materials can be found at https://www.mdpi.com/1422-0067/21/23/8917/s1.

## Abbreviations

AS	aortic stenosis
AVIC	aortic valve interstitial cell
CAVD	calcific aortic valve disease
CN	calcific nodule
COX-2	cyclooxygenase-2
SMA	smooth muscle actin
TGF	transforming growth factor
