# Supplementary material for: COX-2 Is Downregulated in Human Stenotic Aortic Valves and Its Inhibition Promotes Dystrophic Calcification
Source: Int J Mol Sci. 2020 Nov 24;21(23):8917. doi: 10.3390/ijms21238917 (PMC7727817; doi:10.3390/ijms21238917)
Supplement: Supplementary file 1 [file ijms-21-08917-s001.pdf]

Supplementary data

Supplementary Table S1. Correlation between COX-2 expression and clinical characteristics in CAVD patients. BMI, body mass index; eGFR, estimated glomerular filtration rate; LDL, low density lipoprotein; LV, left ventricle; EDVi/ESVi, end diastolic/systolic volume index; EF, ejection fraction; LVMI, left ventricle mass index; AV, aortic valve; MPG, mean pressure gradient. Correlation between COX-2 expression and clinical variables were performed with non-parametrical Spearman correlation.

**Correlation between COX-2 expression and clinical characteristics**	**Spearman coefficient**	**p**
Age (years)	-0.048	0.738
BMI (kg/m^2)	0.235	0.094
**Laboratory data**		
Hemoglobin (g/dl)	-0.139	0.325
Platelets ($*10^3/mm^3$)	-0.065	0.647
Glucose (mg/dl)	-0.064	0.653
eGFR (ml/min)	0.143	0.311
LDL (mg/dl)	0.123	0.394
Albumin (g/dl)	0.112	0.434
**Echocardiography data**		
LVEDVi (ml/m^2)	0.141	0.339
LV ESVi (ml/m^2)	0.112	0.443
LV EF (%)	-0.080	0.575
LVMI (g/m^2)	0.045	0.784
AV MPG (mmHg)	-0.070	0.623
AV peak velocity (m/s)	-0.077	0.589

Supplementary Table S2. Characteristics of AVICs donors

Sample #	Gender	Age	Diagnosis
1	M	65	Ascending aortic aneurysm with severe aortic regurgitation
2	F	68	Severe aortic regurgitation due to prior endocarditis
3	F	73	Ascending aortic aneurysm with severe aortic regurgitation
4	M	70	Aortic valve prolapse with severe aortic regurgitation
5	M	68	Severe aortic stenosis (CAVD)
6	F	69	Severe aortic stenosis (CAVD)
7	F	79	Severe aortic stenosis (CAVD)
8	F	80	Severe aortic stenosis (CAVD)
9	F	71	Severe aortic regurgitation

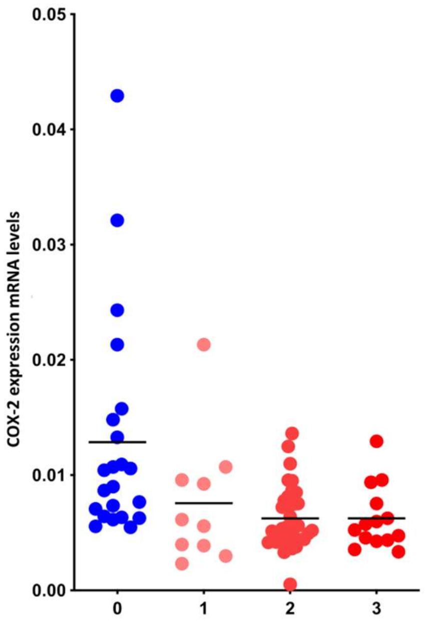

Supplementary Figure S1. COX-2 expression in aortic valve with different degree of calcification. (A) Levels of COX-2 mRNA in non-calcific AVs (calcification score 0; blue dots) or CAVD with different degrees of calcification (calcification score 1-3; red dots). Calcification scores were assigned as follows: 0) No thickening, if present, nodules less than 2mm; 1) Mild thickening 1-2mm, presence of small nodules 2-4mm; 2) Moderate thickening 2-4mm, severe calcification; 3) Severe thickening 5-10mm; extensive calcification.

Supplementary Methods

Materials

The antibody to COX-2 was from Abcam (Cambridge, UK). Fetal bovine serum (FBS), collagenase II, HBSS, PBS, penicillin/streptomycin, M199 medium, SuperScript III reverse transcriptase, random hexamers, dNTPs, RNaseOut, primers for RT-PCR, RIPA buffer, LDS sample buffer, sample reducing agent, peroxidase-conjugate secondary antibodies, Annexin V-FITC and Propidium iodide were from ThermoFisher Scientific (Waltham, MA, USA). Detergent compatible (DC) protein assay, mini-PROTEAN® TGX™, non-fat dry milk, Trans-Blot Turbo Transfer Starter System, Clarity and Clarity Max Western ECL were from BioRad (Hercules, CA, USA). RNeasy Fibrous Tissue Mini Kit was from Qiagen (Hilden, Germany). PerfeCta SYBR Green SuperMix with ROX kit were from Quanta Biosciences (Gaithersburg, MD, USA). Alizarin Red, phenylmethylsulfonyl fluoride (PMSF), PFA, Tween20, BSA, TGF- β1, celecoxib, FGF-2, insulin, ascorbic acid, dexamethasone, β-glycerophosphate and the mouse monoclonal antibody to α-SMA and β-actin (AC-15) were purchased from Sigma Aldrich (St. Louis, MO, USA). Phosphatase inhibitor (PhosSTOP) was from Roche. Protease inhibitors Mix was purchased from SERVA (Heidelberg, Germany). Movat pentachrome stain Kit (modified Russell-Movat) was from Abcam (Cambridge, UK).

Gene expression

Aortic valves leaflets removed during surgery were immediately immersed in RNAlater (ThermoFisher Scientific, Waltham, Massachusetts, USA). Specimens were then disrupted and homogenized with the TissueRuptor® (Qiagen, Hilden, Germany) and total RNA extracted with RNeasy® Fibrous Tissue Mini Kit (Qiagen, Hilden, Germany) according to the manufacturer's instruction. RNA concentration and purity were evaluated by Nanodrop 2000 spectrophotometer (ThermoFisher Scientific). 100 ng of RNA were reverse transcribed in a volume of 25 μl using 250 units of SuperScript III reverse transcriptase and 50 ng of random hexamers on SimpliAmp™ Thermal Cycler (ThermoFisher Scientific). 2 μl of the cDNA mixture were used

for real-time PCR experiments. Real-time PCR reactions were conducted on StepOnePlus™ Real-Time PCR System (ThermoFisher Scientific), using PerfeCta SYBR Green SuperMix with ROX kit (Quanta Biosciences, Beverly, MA, USA) according to the manufacturer's protocol. Primers concentration was 500 nM. Primers sequences used are: RPL13: forward 5′-GGAGGTGCAGGTCCTGGTGCTT-3′, reverse 5′-CGTACGACCACCACCTTCCGG-3′; PTGS2 (COX2): forward 5′-CAAATTGCTGGCAGGGTTGC-3′, reverse 5′-AGGGCTTCAGCATAAAGCGT-3′; ACTA-2 (α-SMA).: forward 5′-CCGACCGAATGCAGAAG-3′, reverse 5′-ACAGAGTATTTGCGCTCCGAA-3′. 3 non-calcific samples was not used for α-SMA expression due to limited amount of RNA and/or cDNA. Gene expression levels were expressed as relative copy number calculated with $2^{-\Delta Ct}$ formula using RPL13 as reference gene.

AVIC isolation and cell culture

Leaflets were incubated with 600 U/mL collagenase II (ThermoFisher Scientific) for 10 minutes at 37°C then were gently scraped to remove aortic valve endothelial cells. Leaflets were then rinsed with HBSS and then diced before incubation with 200 U/mL collagenase II for 4 hours to disaggregate the tissue. Next, the cell suspension was repeatedly pipetted, and filtered through a 100 μm cell strainer to remove residual chunks and spun at 400 g to obtain a cell pellet that was washed with HBSS. Finally, cells were plated and grown in M199 medium (ThermoFisher Scientific) containing 2% FBS (ThermoFisher Scientific) and 1% penicillin/streptomycin (ThermoFisher Scientific). To minimize AVICs trans-differentiation to myofibroblasts which may occur during culturing, we added to the medium 10 ng/ml of Fibroblast Growth Factor (FGF) 2 and 50 ng/ml insulin (Sigma Aldrich) (Porras et al. 2017; Latif et al. 2015). To induce AVICs calcification cells were grown in M199 medium supplemented with 10% FBS, 50 μM ascorbic acid, 0.1 μM dexamethasone and 10 mM β-glycerophosphate (Sigma Aldrich) for 14 days. AVICs were used between passages 3 and 5.

Histology and Immunohistochemistry

For histological and immunofluorescence analysis, aortic valves removed during surgery were immediately fixed in 10% neutral buffered formalin for 24h and then embedded in paraffin. For histology and

immunostaining, 5 μm sections were cut using a microtome (SLEE medial, Mainz, Germany) and placed on polylysine glass slides. For histology, sections were re-hydrated and stained with Movat's pentachrome stain (Abcam, Cambridge, UK) according to manufacturer's instructions. For immunofluorescence analysis, sections were re-hydrated and antigen retrieval was performed in 10mM citrate buffer at 95 °C for 13 minutes. Sections were washed with PBS for 20 minutes and then blocked and permeabilized with 10% BSA solution with 0.3% Triton-X 100 for 1 hour. After blocking, sections were washed 3 times with PBS 1X and then incubated at 4 °C overnight with rabbit anti-COX2 and mouse α-SMA antibodies diluted in 1% bovine serum albumin (BSA). Unbound antibody was removed washing 3 times with PBS and sections were incubated for 1 hour at room temperature in the dark with anti-rabbit antibody conjugated with Alexa Fluor 488 and anti-mouse antibody conjugated to and Alexa Fluor 647 (ThermoFisher Scientific) diluted in 1% BSA. After 3 washes in PBS 1X, slides were mounted with aqueous mounting solution containing DAPI Prolong (ThermoFisher Scientific). Images were taken with a confocal microscope (Nikon A1 system) using 10X or 20X objectives.

Apoptosis

Apoptosis was evaluated with the Annexin V/ Propidium Iodide (PI) binding assay. After treatments, AVICs were stained with Annexin V-FITC and PI (ThermoFisher Scientific) in a binding buffer (10 mM Hepes, 5 mM KCl, 150 mm NaCl, 1.8 mM $CaCl_2$, 1 mM $MgCl_2$, pH 7.4), left in the dark at room temperature for 20 min. Annexin V-FITC and PI positive cells were quantified by flow cytometry (Attune Nxt Flow cytometer, ThermoFisher Scientific). The data were analyzed with Attune Nxt Software (ThermoFisher Scientific). Annexin V-positive cells were considered apoptotic, and apoptosis was expressed as percentage of Annexin V-positive cells on total cells.
